# Analgesic purchases among older adults – a population-based study

**DOI:** 10.1186/s12889-021-10272-3

**Published:** 2021-01-31

**Authors:** Maiju K. Marttinen, Hannu Kautiainen, Maija Haanpää, Heini Pohjankoski, Jukka Hintikka, Markku J. Kauppi

**Affiliations:** 1 Faculty of Medicine and Health Technology, Tampere University, Arvo Ylpön katu 34, 33520 Tampere, Finland; 2 Department of Anesthesiology, Intensive Care and Pain Medication, University of Helsinki and Helsinki University Hospital, Haartmaninkatu 8, 00290 Helsinki, Finland; 3 Folkhälsan Research Center, Topeliuksenkatu 20, 00250 Helsinki, Finland; 4 Primary Health Care Unit, Kuopio University Hospital, Puijonlaaksontie 2, 70200 Kuopio, Finland; 5 Ilmarinen Mutual Pension Insurance Company, Porkkalankatu 1, 00180 Helsinki, Finland; 6 Department of Neurosurgery, Helsinki University Hospital, Topeliuksenkatu 5, 00270 Helsinki, Finland; 7 Department of Paediatrics, Päijät-Häme Central Hospital, Keskussairaalankatu 7, 15850 Lahti, Finland; 8 Department of Psychiatry, Päijät-Häme Central Hospital, Keskussairaalankatu 7, 15850 Lahti, Finland; 9 Department of Rheumatology, Päijät-Häme Central Hospital, Keskussairaalankatu 7, 15850 Lahti, Finland

**Keywords:** Analgesic, NSAID, Opioid, Elderly, Pain

## Abstract

**Background:**

Pain is a frequent and inevitable factor affecting the quality of life among older people. Several studies have highlighted the ineffectiveness of treating chronic pain among the aged population, and little is known about the prevalence of analgesics administration among community-dwelling older adults. The objective was to examine older adults’ prescription analgesic purchases in relation to SF-36 pain in a population-based setting.

**Methods:**

One thousand four hundred twenty community-dwelling citizens aged 62–86 years self-reported SF-36 bodily pain (pain intensity and pain-related interference) scores for the previous 4 weeks. The Social Insurance Institution of Finland register data on analgesic purchases for 6 months prior to and 6 months after the questionnaire data collection were considered. Special interest was focused on factors related to opioid purchases.

**Results:**

Of all participants, 84% had purchased prescription analgesics during 1 year. NSAIDs were most frequently purchased (77%), while 41% had purchased paracetamol, 32% opioids, 17% gabapentinoids, and 7% tricyclic antidepressants. Age made no marked difference in purchasing prevalence. The number of morbidities was independently associated with analgesic purchases in all subjects and metabolic syndrome also with opioid purchases in subjects who had not reported any pain.

**Discussion:**

Substantial NSAID and opioid purchases emerged. The importance of proper pain assessment and individual deliberation in terms of analgesic contraindications and pain quality, as well as non-pharmacological pain management, need to be highlighted in order to optimize older adults’ pain management.

## Background

Chronic pain is one of the leading conditions in later life in terms of commonness and economic burden [1]. To manage pain in older adults is known to be complex and should always be based on proper assessment [2]. Despite the commendable development of pain management in recent years, the overall consensus highlights the under-assessment, under-diagnosing, and under-treatment/mistreatment of persistent pain in older individuals [3, 4].

Successful pain management is based on balancing the benefits and harms of available drugs, on lifestyle interventions, and on treating the underlining cause as much as possible [5]. Paracetamol has been recommended as the first-line treatment for both acute and chronic pain in older people, followed by non-steroidal anti-inflammatory drugs (NSAIDs), but only if contraindications are not present [6, 7]. Pharmacological treatment modalities need to be combined with non-pharmacological ones [6, 7]. Opioid administration may be considered with moderate to severe cancer and non-cancer pain, but never without precise individual deliberation and careful monitoring [7, 8]. Long-term opioid administration for chronic non-cancer pain remains controversial [9].

It has been discussed that NSAID use does not adhere to clinical guidelines (e.g. long-term or pro re nata use despite contraindications) in older adults [10–13], among whom NSAIDs have been suggested to comprise a major cause of drug-related morbidity [14]. NSAID-related peptic ulcers, renal and cardiovascular adverse effects, and increased mortality have been reported [14, 15]. Age-related physiological changes, cognitive impairment (assessment, adverse effects), multi-morbidity, and polypharmacy pose major challenges to opioid administration [6, 16]. Adverse effects are relatively frequent, with potentially severe consequences [16]. An appropriate dose needs to be carefully titrated and both the desired and adverse effects monitored regularly [7, 8, 17].

Studies regarding analgesic administration among community-dwelling older adults are scarce [10, 13, 18, 19]. It remains unclear how large a proportion of older adults actually use analgesics and whether the medication consists of NSAIDs or paracetamol, or of neuropathic drugs or opioids [11, 20]. Multiple studies have suggested an increasing trend in opioid prescribing over the recent years [21, 22], but little is known about the actual opioid administration among older adults [7, 19, 23].

The two main research questions of the current study were as follows: 1) What are the purchasing prevalence and profile of use of separate analgesics among older community-dwelling citizens? 2) What is the relationship between reported SF-36 pain and use of different analgesics among older adults?

Based on the clinical point of view and previous studies that have presented an increasing trend in opioid prescribing over the past two decades [22], opioid administration was expected to emerge as relatively extensive. To identify factors related to opioid use could prove beneficial in terms of prevention. Therefore, the third research question was: 3) Which factors are related to opioid purchases?

## Methods

The current research was a part of the Good Ageing in Lahti Region (GOAL) study executed in the Päijät-Häme Hospital District in Southern Finland [24]. The district consists of both rural and urban areas, with approximately 220,000 inhabitants. The GOAL was a 10-year follow-up cohort study (*N* = 2815) that introduced a stratified (age, sex, 14 municipalities) random sample of Finnish seniors born in 1926–30, 1936–40, and 1946–50. At baseline (2002) and the three follow-up visits (2005, 2008, and 2012), an extensive questionnaire was filled out (overall health, attitudes, quality of life, etc.), and blood samples and physical data were collected. The design of the study has been described in more detail elsewhere [24]. The present study focused on the 2012 data, which included the complete data regarding prescribed pain medications. The total number of participants still attending at this point of the follow-up was *N* = 1697. Subjects with insufficient data regarding reported pain (*N* = 277) were excluded, and all statistical analyses were therefore executed with 1420 subjects. In 2012, the participants were 62–-66, 72–76, and 82–86 years of age. Hospitalized and institutionalized older adults did not participate in the study.

In Finland, patients are entitled to a reimbursement of medication costs from the Social Insurance Institution of Finland (SII). SII maintains a nationwide register of all prescriptions and medication purchases, from which the present data regarding analgesic purchases were retrieved. All pain medication purchases 6 months prior to and 6 months after the questionnaire data collection were considered. The presence vs absence of the drugs in the participants’ purchasing history were retrieved. The exact number and dose were not retrievable. The temporal association between the pain assessment and analgesic purchases was not retrievable. The analgesics considered were level 1 analgesics (NSAIDs [Anatomical Therapeutic Chemical Classification System (ATC)] M01AE01, M01AE51 ibuprofen; M01AE03 ketoprofen; M01AH01, M01AH05, M01AH06 COX-2 selective inhibitors; M01AC06 meloxicam; M01AE02, M01AE52 naproxen; M01AB05 diclofenac; M01AB01 indometin]; as well as N02BE01, N02BE51 paracetamol) and level 2–3 analgesics (N02AA01 morphine; N02AA03 hydromorphone; N02AA05 oxycodone; N02AA55 oxycodone-naloxone; N02AA59, N02AJ06 codeine combinations; N02AB03 fentanyl; N02AE01 buprenorphine; N02AX02, N02AJ14 tramadol and tramadol combinations), in addition to gabapentinoids (N03AX23 gabapentin; N03AX16 pregabalin) and tricyclic antidepressants (TCAs [N06AA09, N06CA01 amitriptyline; N06AA10 nortriptyline]). Acetylsalicylic acid was excluded from the NSAIDs due to its major use as an antithrombotic drug.

Data on the study participants’ pain, demographics, life habits, morbidity, and symptoms were based on the GOAL questionnaire. Regarding pain, the two-item Bodily Pain section of the SF-36 questionnaire was used [25, 26]. The participants indicated how severe pain they had experienced during the previous 4 weeks (intensity; 100 = none, 80 = very mild, 60 = mild, 40 = moderate, 20 = severe, 0 = very severe) and how much this pain had disrupted their everyday work and activity (at home or outside of home) during the previous 4 weeks (interference; 100 = not at all, 75 = a little bit, 50 = moderately, 25 = quite a bit, 0 = extremely). Bodily pain is presented herein as the mean of the pain intensity and interference scores. In the first analysis, based on the bodily pain scores, the subjects were divided into four pain groups (*group I* [0–45, moderate to very severe pain intensity and interference], *group II* [47.5–70], *group III* [77.5–90], *group IV* [100, no pain intensity and interference at all]). The groups were established in order to be able to combine pain intensity and pain-related interference and to consider analgesic administration in relation to the pain. The rationale for the four pain groups was to consider separately the subjects who had reported high levels of both pain intensity and pain interference (*I*) and those who had reported none (*IV*). The rest were divided into two groups (*II–III*) to equate the group sizes. In the second analysis, subjects were divided into two groups (opioid, non-opioid) based on whether they had purchased opioids.

Household income was determined as the taxable household income divided by the square root of the number of people living in the household indexed to the year 2017 [27]. Weekly alcohol consumption was measured with the 3-item AUDIT-C [28] instrument. Leisure-time physical activity (LTPA) was determined as activities lasting over 30 min that make the participant sweat and pant at least to some degree (high [6–7 times a week], moderate [3–5 times a week], low [1–2 times a week or less, or not possible due to injury or illness]) [29]. The provided laboratory test data (fP-Glucose, fP-Triglyceride, high-density lipoprotein [fP-HDL], estimated glomerular filtration rate [eGFR, ml/min/1.73 m2], high-sensitivity C-reactive protein [S-hs-CRP], rheumatoid factor, serum uric acid) and clinical measurements (weight, height, blood pressure, waist circumference) were considered. The morbidities examined included cardiovascular, pulmonary, musculoskeletal, psychiatric, and neurological diseases, as well as diabetes mellitus type II and neoplasms. Prior to the regression analyses, the sum of each participant’s diagnosed morbidities was calculated. Metabolic syndrome (MetS) was determined as the presence of three or more of the following components: 1) waist circumference ≥ 102 cm for men and ≥ 88 cm for women; 2) fP-Triglycerides ≥1.7 mmol/L or treatment for dyslipidemia; 3) fP-HDL ≤1.03 mmol/L for men and ≤ 1.29 mmol/L for women, or treatment for dyslipidemia; 4) systolic blood pressure ≥ 130 mmHg or diastolic blood pressure ≥ 85 mmHg, or antihypertensive medication; and 5) fP-Glucose ≥5.6 mmol/L or the use of medication for hyperglycemia [30]. Also, the number of doctor’s appointments during the previous 12 months was considered. For more details about variables and coding, please contact the corresponding author for the GOAL Codebook.

The descriptive statistics include means and SDs for continuous variables and numbers and percentages for categorical variables. Statistical significances for the hypothesis of linearity across the SF-36 categories of bodily pain were evaluated by using the Cochran–Armitage test for trend and analysis of variance with an appropriate contrast. Statistical comparisons between the opioid usage groups were performed with the t-test, the Chi-squared test, or the Fisher-Freeman-Halton test when appropriate. In the case of a violation against the assumptions (non-normality), a bootstrap-type test was applied. Multivariate logistic regression was employed to investigate factors related to analgesic purchases. As predictors, the following were included: pain levels and LTPA (as ordinal variables); sex, MetS, and smoking (as dicotomous variables); and age, education years, Audit-C and number of morbidities (as continuous variable). The Hosmer-Lemeshow goodness-of-fit statistics were used for the assessment of the final models. The normality of variables was evaluated with the Shapiro-Wilk W test. The Stata 15.1 statistical package by StataCorp LP (College Station, TX, USA) was used for the analyses.

## Results

The mean age of all 1420 participants was 71.2 years. The proportion of females was 55%. Out of the participants, 84% had purchased some prescribed analgesics during the considered year. All of the prescribed analgesics were most frequently obtained by *group I* subjects, who reported the most pain, with the percentage decreasing linearly with the groups. In *group I*, 91% of the participants had picked up the prescribed NSAIDs. NSAIDs were also largely purchased by those who, at the moment of the questionnaire data collection, reported no SF-36 bodily pain at all (70%). Forty-one percent of all participants had purchased prescribed paracetamol. Over half of the participants in *group I* had purchased the prescribed opioids, as had almost one fourth of those with no reported pain at all. In *group I*, 17% had purchased gabapentinoids and 7% TCAs. Age (62–66 vs 72–76 vs 82–86 years) did not make a marked difference in drug distribution or purchasing prevalence. The analgesic purchases in relation to the four SF-36 bodily pain and according to three age groups are presented in Table 1.

**Table 1:** Prescribed analgesics according to four SF-36 bodily pain levels (*groups I–IV*)

Pain group	I N (%)	II N (%)	III N (%)	IV N (%)	***P***-value*
ALL PARTICIPANTS					
Number	244	382	478	316	
Level 1 analgesics	225 (92)	323 (85)	392 (82)	234 (74)	< 0.001
NSAID	200 (91)	303 (80)	375 (78)	222 (70)	< 0.001
Paracetamol	151 (62)	185 (48)	162 (34)	85 (27)	< 0.001
Level 2–3 analgesics	127 (52)	133 (35)	127 (27)	73 (23)	< 0.001
Mild opioid	123 (50)	131 (34)	124 (26)	71 (22)	< 0.001
Intermediate/strong opioid	24 (10)	9 (2)	5 (1)	4 (1)	< 0.001
Neuropathic pain medication					
Gabapentinoid	42 (17)	34 (9)	23 (5)	13 (4)	< 0.001
Tricyclic antidepressant	16 (7)	15 (4)	14 (3)	5 (2)	< 0.001
AGE 62–66 YEARS					
Number	71	163	224	145	
Level 1 analgesics	66 (93)	133 (82)	177 (79)	110 (76)	< 0.001
NSAID	66 (93)	129 (79)	176 (79)	109 (75)	< 0.001
Paracetamol	35 (49)	58 (36)	56 (25)	32 (22)	< 0.001
Level 2–3 analgesics	41 (58)	51 (31)	60 (27)	31 (21)	< 0.001
Mild opioid	40 (56)	50 (31)	59 (26)	29 (20)	< 0.001
Intermediate/strong opioid	4 (6)	2 (1)	2 (1)	2 (1)	< 0.001
Neuropathic pain medication					
Gabapentinoid	12 (17)	10 (6)	14 (6)	5 (3)	< 0.001
Tricyclic antidepressant	7 (10)	7 (4)	9 (4)	0 (0)	0.016
AGE 72–76 YEARS					
Number	112	150	197	129	
Level 1 analgesics	102 (91)	132 (88)	168 (85)	92 (71)	< 0.001
NSAID	99 (88)	123 (82)	158 (80)	86 (67)	< 0.001
Paracetamol	75 (67)	83 (55)	79 (40)	33 (26)	< 0.001
Level 2–3 analgesics	56 (50)	56 (37)	51 (26)	34 (26)	< 0.001
Mild opioid	54 (48)	56 (37)	49 (25)	34 (26)	< 0.001
Intermediate/strong opioid	12 (11)	5 (3)	3 (2)	1 (1)	< 0.001
Neuropathic pain medication					
Gabapentinoid	20 (18)	17 (11)	8 (4)	4 (3)	< 0.001
Tricyclic antidepressant	6 (5)	5 (3)	3 (2)	5 (4)	0.023
AGE 82–86 YEARS					
Number	61	69	57	42	*
Level 1 analgesics	57 (93)	58 (84)	47 (82)	32 (76)	< 0.001
NSAID	56 (92)	54 (78)	41 (72)	27 (64)	< 0.001
Paracetamol	41 (67)	44 (64)	27 (47)	20 (48)	< 0.001
Level 2–3 analgesics	30 (49)	26 (38)	16 (28)	8 (19)	< 0.001
Mild opioid	29 (48)	25 (36)	16 (28)	8 (19)	< 0.001
Intermediate/strong opioid	8 (13)	2 (3)	0 (0)	1 (2)	< 0.001
Neuropathic pain medication					
Gabapentinoid	10 (16)	7 (10)	1 (2)	4 (10)	< 0.001
Tricyclic antidepressant	3 (5)	3 (4)	2 (4)	0 (0)	0.019

^*^p for linearity. Groups I–IV: *group I* [0–45, moderate to very severe pain intensity and interference], *group II* [47.5–70], *group III* [77.5–90], *group IV* [100, no pain intensity and interference at all]. Three age groups: 62–66, 72–76, 82–86 years

In total, 32% of the participants had purchased opioids. Between the opioid and non-opioid group, only a moderate difference was found in SF-36 pain intensity (61 in the opioid group vs 72 in the non-opioid group) and interference (71 vs 82, respectively) (*p* < 0.001). Cardiovascular, musculoskeletal, and pulmonary diseases, as well as neoplasms, musculoskeletal pain, and depressive symptoms were more prevalent among opioid users. MetS was present in 47% of the participants in the opioid group, compared to the corresponding 40% in the non-opioid group (*p* = 0.009). The proportion of those with a BMI of over 30 was higher in the opioid group (*p* = 0.049). Among male participants, waist circumference was slightly wider in those who were on opioids. Serum hs-CRP and uric acid levels were higher among the participants in the opioid group. Furthermore, 48% of the participants in the opioid group had visited a doctor more than 3 times during the previous 12 months, compared to the corresponding 29% in the non-opioid group (*p* < 0.001). No differences were found between the groups in terms of cohabiting, education years, household income, LTPA, waist circumference in females, alcohol consumption, smoking, blood pressure, prevalence of diabetes mellitus type II, psychiatric or neurological disease, headache, insomnia, or other laboratory measurements. Table 2 illustrates the characteristics of the groups and all related factors examined.

**Table 2:** Characteristics of 1420 GOAL participants who had (Yes) or had not (No) used opioids during one year

	Opioids purchased		
	No *N* = 960	Yes *N* = 460	*P*-value
Pain			
Bodily Pain, mean (SD)	77 (21)	66 (25)	< 0.001*
Intensity, mean (SD)	72 (23)	61 (26)	< 0.001*
Interference, mean (SD)	82 (22)	71 (27)	< 0.001*
Demographics			
Female sex, n (%)	534 (56)	250 (54)	0.65
Age, mean (SD)	71 (7)	72 (7)	0.16
Cohabiting, n (%)	662 (69)	321 (70)	0.75
Education years, mean (SD)	9.8 (3.2)	9.6 (3.1)	0.37
OECDsgrt^a^, mean (SD) 1000€	1.8 (1.1)	1.7 (0.6)	0.40
Smoking, n (%)	141 (15)	68 (15)	0.97
AUDIT-C^b^, mean (SD)	2.6 (2.2)	2.6 (2.2)	0.83
LTPA^c^, n(%)			0.24
Low	118 (12)	71 (15)	
Moderate	689 (72)	323 (71)	
High	148 (16)	64 (14)	
Clinical			
BMI^d^, mean (SD)	27.8 (4.8)	28.4 (4.7)	0.021*
Obese (BMI^d^ ≥ 30), n (%)	241 (26)	139 (31)	0.049*
Waist cm, mean (SD)			
Female	92 (14)	94 (13)	0.057
Male	100 (11)	103 (11)	0.002*
MetS^e^, n (%)	385 (40)	218 (47)	0.009*
Blood pressure mmHg, mean (SD)			
Systolic	138 (21)	137 (19)	0.32
Diastolic	83 (11)	82 (10)	0.10
Morbidity (diagnosed), n (%)			
Cardiovascular disease	413 (43)	225 (49)	0.037*
Diabetes mellitus type II	101 (11)	56 (12)	0.35
Musculoskeletal disease	330 (34)	231 (50)	< 0.001*
Pulmonary disease	69 (7)	55 (12)	0.003*
Psychiatric disease	27 (3)	22 (5)	0.057
Neurological disease	25 (3)	16 (6)	0.36
Neoplasm	47 (5)	44 (10)	< 0.001*
Symptoms, n (%)			
Joint pain	264 (28)	182 (40)	< 0.001*
Back pain	235 (24)	163 (35)	< 0.001*
Neck pain	253 (26)	157 (34)	< 0.001*
Headache	109 (11)	61 (13)	0.30
Insomnia	176 (18)	91 (20)	0.51
Depression	40 (4)	32 (7)	0.025*
Laboratory tests, mean (SD)			
Glucose, mMol/L	5.66 (1.05)	5.68 (1.01)	0.72
Triglyceride, mMol/L	1.26 (0.65)	1.23 (0.59)	0.47
HDL, mMol/L	1.58 (0.46)	1.55 (0.47)	0.35
eGFR, mL/min/1.73 cm3	71 (14)	71 (15)	0.89
hsCRP, mg/L	4.2 (4.0)	5.4 (9.5)	0.001*
Uric acid, uMol/L	334 (73)	343 (79)	0.042*
Visited physician ≥3 times, n (%)	282 (29)	223 (48)	< 0.001*

*P-values less than 0.05 were considered statistically significant. Data are presented as mean values (SD) and as absolute values and percentages. ^a^*OECDsgrt* OECD square root–determined household income; ^b^*AUDIT-C* Alcohol unit consumption per week; ^c^*LTPA* Leisure-time physical activity; ^d^*BMI* Body mass index; ^e^*MetS* Metabolic syndrome. Laboratory test abbreviations: *HDL* High-density lipoprotein, *eGFR* Estimated glomerular filtration rate, *hsCRP* High-sensitivity C-reactive protein

According to logistic regression, only pain level and a higher number of morbidities were found to independently associate with purchases of Level 1 (NSAIDs and paracetamol; 1.30 [1.04 to 1.63], *p* = 0.020) and Level 2–3 analgesics (opioids; 1.53 [1.30 to 1.79], *p* < 0.001). No significant association was found between analgesic purchases and the following variables: sex, age, education years, smoking, alcohol consumption, LTPA, or MetS (Table 3).

**Table 3:** Analgesic purchases and associated factors in 1420 GOAL participants. A multivariate logistic regression model

	Analgesic purchases			
	Level 1 analgesics		Level 2–3 analgesics	
	OR (95% CI)	*p*-value	OR (95% CI)	*p*-value
Pain levels^a^		< 0.001*		< 0.001*
*Group I*	1.00 (Reference)		1.00 (Reference)	
*Group II*	0.54 (0.31 to 0.93)		0.56 (0.40 to 0.79)	
*Group III*	0.48 (0.28 to 0.82)		0.41 (0.29 to 0.58)	
*Group IV*	0.30 (0.17 to 0.53)		0.36 (0.24 to 0.53)	
Male sex	0.74 (0.53 to 1.01)	0.060	1.13 (0.86 to 1.48)	0.38
Age	1.01 (0.99 to 1.03)	0.45	0.99 (0.97 to 1.01)	0.36
Education years	1.01 (0.97 to 1.06)	0.56	1.01 (0.97 to 1.04)	0.78
Smoking	1.21 (0.78 to 1.86)	0.39	1.03 (0.72 to 1.46)	0.87
AUDIT-C^b^	1.00 (0.93 to 1.08)	0.92	1.00 (0.94 to 1.07)	0.99
LTPA^c^		0.39		0.34
Low	1.00 (Reference)		1.00 (Reference)	
Moderate	1.33 (0.88 to 2.00)		0.86 (0.61 to 1.22)	
High	1.27 (0.76 to 2.14)		0.80 (0.51 to 1.25)	
MetS^d^	0.91 (0.67 to 1.22)	0.52	1.09 (0.86 to 1.40)	0.47
Number of morbidities^e^	1.30 (1.04 to 1.63)	0.020*	1.53 (1.30 to 1.79)	< 0.001*

*P-values less than 0.05 were considered statistically significant. Level 1 analgesics: paracetamol, NSAIDs; Level 2–3 analgesics: weak to strong opioids. ^a^Pain levels: Groups I–IV: *group I* [0–45, moderate to very severe pain intensity and interference], *group II* [47.5–70], *group III* [77.5–90], *group IV* [100, no pain intensity and interference at all]; ^b^*AUDIT-C* Alcohol unit consumption per week, ^c^*LTPA* Leisure-time physical activity, ^d^*MetS* Metabolic syndrome, ^e^*Number of morbidities* sum of each participant’s diagnosed morbidities

At the time of the questionnaire data collection, 16% of the participants in the opioid group reported no SF-36 pain of any intensity and 30% no SF-36 pain-related interference. Of all examined variables presented in Table 4, in addition to morbidities, MetS was the only factor to independently associate with opioid administration among these subjects (OR_intensity_ 1.99 [CI 1.10–3.60, *p* = 0.022], OR_interference_ 1.60 [CI1.05–2.43, *p* = 0.029]).

**Table 4:** Associated factors in opioid-purchasing participants with no SF-36 pain. A multivariate logistic regression model

	No SF-36 pain intensity *N* = 74		No SF-36 pain-related interference *N* = 138	
	OR (95% CI)	*p*-value	OR (95% CI)	*p*-value
Male sex	0.88 (0.47 to 1.63)	0.68	1.20 (0.78 to 1.86)	0.40
Age	0.99 (0.94 to 1.03)	0.58	0.97 (0.94 to 1.01)	0.095
Education years	1.01 (0.92 to 1.10)	0.88	1.03 (0.97 to 1.09)	0.37
Smoking	1.71 (0.77 to 3.80)	0.19	1.08 (0.62 to 1.90)	0.78
AUDIT-C^a^	0.95 (0.82 to 1.10)	0.47	0.97 (0.88 to 1.08)	0.62
LTPA^b^		0.99		0.64
Low	1 (Reference)		1 (Reference)	
Moderate	0.99 (0.43 to 2.32)		0.92 (0.50 to 1.69)	
High	1.00 (0.34 to 2.87)		0.83 (0.39 to 1.79)	
MetS^c^	1.99 (1.10 to 3.60)	0.022*	1.60 (1.05 to 2.43)	0.029*
Number of morbidities^d^	1.84 (1.17 to 2.90)	0.008*	1.50 (1.08 to 2.07)	0.015*

*P-values less than 0.05 were considered statistically significant. ^a^*AUDIT-C* Alcohol unit consumption per week, ^b^*LTPA* Leisure-time physical activity, ^c^*MetS* Metabolic syndrome, ^d^*Number of morbidities* sum of each participant’s diagnosed morbidities. Comparison to subjects that had received opioid prescription and reported some pain 1) intensity for ‘No pain intensity’ 2) interference for ‘No pain interference’

## Discussion

The present population-based study examined prescription analgesic purchases among older adults in a modern Western society. As a major finding, analgesic purchases were revealed to be substantial. Drug distribution and purchasing prevalence did not markedly differ between three age groups (62–66, 72–76 and 82–86 years).

### Analgesic purchases

It has been debated whether medical professionals are lacking in courage when it comes to sufficient pharmacological pain treatment in older adults [31, 32]. The results herein indicate that analgesics are in substantial use. The prevalence of analgesic purchases was highest in *group I.* This was expected, although no temporal association between the pain assessment and analgesic purchases was not retrievable. However, analgesics were also purchased by subjects who, at the time of questionnaire data collection, had reported little or no pain.

The major findings of the current study were the abundant purchases of NSAIDs and opioids, and the relatively minor purchases of paracetamol and neuropathic analgesics. The majority of seniors had purchased at least one prescribed package of NSAIDs during 1 year, which may be regarded as concerning, considering the multiple potential contraindications to NSAIDs in older adults. Surprisingly, paracetamol purchases were in a clear minority compared to NSAIDs, despite it being recommended as the drug-of-choice in older adults’ pain management [6, 7]. Recently, physicians have been encouraged to consider not prescribing paracetamol for patients with lower back pain and osteoarthritis due to possible inefficacy [33]. It is most likely that a major part of older adults’ pain is due to musculoskeletal and chronic inflammatory diseases, for which NSAIDs are effective [34, 35]. NSAIDs have previously been reported to be the most prevalent analgesic among robust and paracetamol among frail and pre-frail community-dwelling Finnish elderly individuals [18].

On the other hand, the number of those purchasing analgesics may actually be even higher than what is reported herein, when considering the amount purchased over the counter [11]. In Finland, small packages of paracetamol 500 mg and some small-dose NSAIDs (e.g. ibuprofen) are also available in pharmacies without prescription, but larger packages with a prescription are less expensive in long-term use. Yet, regarding older adults, a strong association between increased age and the use of only prescribed analgesics has been found [36].

Neuropathic drugs were relatively underrepresented, although the prevalence of neuropathic pain is known to increase with age [37]. It is possible that a fear of anticholinergic adverse effects and for example risk of falls and sedation restricted their use. General practitioners are often responsible for community-dwelling older adults’ pain management. They may lack expertise in proper pain assessment and in the use of neuropathic drugs, in addition to having limited time and resources and rarely or never an expert pain management team to refer to. Such a team could most likely be organized relatively easily at least in larger health care centers, if this were considered a priority.

### Opioid administration

The number of prescription opioid purchases emerged as extensive. It is known that opioids are widely used, and the opioid crisis is no longer regarded as a medical, but rather as a public health crisis [38]. In the USA, the number of clinical visits where opioids were prescribed to an older person doubled within a decade (from 1999 to 2000 to 2009–2010) [39]. Pitkälä and colleagues also came to a similar conclusion in their Finnish follow-up study [40]. However, large studies evaluating opioid use among community-dwelling older adults are scarce, estimating a prevalence of 6–9% [18, 41–44]. The results of the present study markedly exceeded what has been reported previously. This may be regarded as a concerning continuation of the previously reported evolution in opioid prescribing, even though the purchases reported herein have most likely been for as-needed or short-term use.

Despite the multiple potential adverse effects, managing pain with opioids may be seen as a tempting choice from the clinicians’ perspective. Opioids are at least relatively effective in almost all pain conditions, and they are not absolutely contraindicated for any patient group. Importantly, the relatively low paracetamol purchasing percentage may also partially be due to substantial opioid consumption; the majority of purchased opioids consisted of mild opioids, which is to say (according to the Finnish practice) a paracetamol-codeine combination or tramadol. Especially for older adults with possible cognitive impairments, clinicians may avoid prescribing concomitant paracetamol and paracetamol-codeine combination due to a fear of patient-related inadequate dose titration and exceeding the paracetamol dose limit. It is possible that a concomitant NSAID and paracetamol-codeine combination has been seen as an effective combination in both acute and chronic pain conditions.

Previously, female sex, poor self-rated health, and the use of over ten non-analgesic drugs have been found to associate with any analgesic use, and moderate and poor self-rated health with opioid use in Finnish community-dwelling older adults over 75 years of age [45]. Interestingly, therein, opioid and daily analgesic use were inversely associated with depressive symptoms [45], whereas in another Finnish study by Gilmartin and colleagues, analgesic use was independently associated with depressive symptoms in patients with Alzheimer’s disease [46]. In the present study, opioid purchases were related to obesity, metabolic syndrome, and morbidities (e.g. depression), while there was no difference between the groups in socioeconomic factors or lifestyle, for instance. There was no trend of purchasing more painkillers among people with a lower socioeconomic status. Also, opioid purchases, as well as NSAID and paracetamol purchases, were independently associated only with a higher number of morbidities. Opioids were purchased by people who had musculoskeletal diseases or neoplasms, which are reasonable causes for the need of opioids. Therefore, the indication for prescribing an opioid has most likely been adequate.

MetS was found to independently associate with opioid purchases in subjects who, at the time of questionnaire data collection, reported no SF-36 pain of any intensity or no pain-related interference. Hyperuricemia and an elevated high-sensitivity C-reactive protein level are often present in MetS [47, 48], and they were both related to opioid purchasing in the present material. It has been shown that an individual with diabetes combined with other MetS components is more likely to have neuropathy [49], and half of all diabetes patients with neuropathy experience neuropathic pain [50]. Interestingly, on the molecular level, severe obesity has recently been found to associate with decreased u-opioid receptor (MOR) availability in the brain [51]. The MORs mediate the effects of both endogenous opioids and exogenous opioid agonists [52]. Decreased MOR availability is known to lead to diminished opioid sensitivity and, further, to opioid tolerance [53]. On the other hand, multiple studies have demonstrated an association between obesity and pain among older people [54, 55]. Further studies are needed to specify whether the MetS/obesity–opioid use association evolves from the molecular level, or whether obesity intensifies musculoskeletal symptoms, for example, thereby leading to a need for opioids. Although the number and doses of opioids prescribed were not retrievable in the present study, clinicians should notice that obese seniors and those with MetS may be more prone to opioid use than older adults in general.

The abundant prescribing of opioids—and of all analgesics—raises the question whether the use of non-pharmacological methods has been sufficient. Almost half of the participants who had purchased opioids had visited a doctor frequently, indicating that these individuals should also have been candidates for non-pharmacological interventions if these had been provided. The role of primary care physicians may be highlighted in evaluating the need for non-pharmacological interventions.

### Strengths and limitations

A setting with self-reports and clinical data combined with Kela-provided data regarding analgesic purchases may be highlighted as the major strength of the present study. The clinical history in relation to analgesic purchases or the exact number and doses of drugs, nor the drugs purchased over the counter or the participants’ other medications were not retrievable, which may be addressed as a limitation. Also, regarding neuropathic pain, duloxetine and venlafaxine prescriptions were not retrievable; however, they were possibly not yet widely used in 2012. In addition, there was a lack of a chronological association between the pain assessment and analgesic purchases. It is possible that the pain was not yet present at the time of the self-report, or, conversely, administered drugs had already relieved the pain, which is why some participants who had purchased analgesics had not reported pain. Additionally, the pattern of prescribing has most likely been based on as-needed use, which has also been reported to be the most common circumstance in previous studies [23], but we cannot ascertain this for certain. Furthermore, drug purchases do not equate with drug use, but investigating the former is the next-best method of estimating the latter. Finally, data used herein was over 5 years old which may be seen as a relative limitation.

## Conclusions

Analgesic administration emerged as substantial in the study, which is comprehensively representative of the Finnish community-dwelling older population. NSAID and opioid purchases were revealed to be extensive, whereas paracetamol and neuropathic analgesics were in the minority. Age made no marked difference in the drug distribution and purchasing prevalence. Number of co-morbidities was the only factor that was independently associated with analgesic purchases in all subjects, and metabolic syndrome also with opioid purchases in subjects who had not reported any bodily pain in the SF-36. Increasing efforts need to be made regarding resources intended for diminishing inappropriate analgesic use in older adults. Future studies are needed to evaluate the consequences (e.g. complications) of extensive use of analgesics in older adults. The significance of proper pain assessment, non-pharmacological pain management (e.g. physical therapy, cognitive behavioral therapy, pain education) and provider based interventions needs to be highlighted.

## Data Availability

The datasets used and/or analyzed during the current study are available from the corresponding author upon reasonable request.

## Abbreviations

AUDIT-C: Alcohol unit consumption per week
BMI: Body mass index
eGFR: Estimated glomerular filtration rate
GOAL: Good Ageing in Lahti Region study
HDL: High-density lipoprotein
hs-CRP: High-sensitivity C-reactive protein
Kela: Social Insurance Institution of Finland
LTPA: Leisure-time physical activity
MetS: Metabolic syndrome
NSAID: Non-steroidal anti-inflammatory drugs
OECDsgrt: OECD square root–determined household income
SF-36: Short-Form 36 questionnaire
TCA: Tricyclic antidepressants
